# Causative Human Papillomavirus (HPV) Genotypes of Anal Cancers in Australian Cisgender Women

**DOI:** 10.1002/jmv.70957

**Published:** 2026-05-08

**Authors:** Clare E. F. Dyer, Monica Molano, Jennifer M. Roberts, Tao Yang, Suzanne M. Garland, Simon Comben, Alyssa M. Cornall, Gerald L. Murray, Richard J. Hillman

**Affiliations:** ^1^ The Kirby Institute, UNSW Sydney Kensington New South Wales Australia; ^2^ Centre for Women's Infectious Diseases, The Royal Women's Hospital Parkville Victoria Australia; ^3^ Douglass Hanly Moir Pathology Sydney New South Wales Australia; ^4^ SydPath, St Vincent's Hospital Sydney New South Wales Australia; ^5^ Murdoch Children's Research Institute Parkville Victoria Australia; ^6^ Department of Obstetrics, Gynaecology and Newborn Health The University of Melbourne Melbourne Victoria Australia; ^7^ St Vincent's Hospital Sydney New South Wales Australia

## Abstract

Anal cancer, caused by persistent infection with oncogenic human papillomavirus (HPV), is rare in the general population. However, certain groups, such as men who have sex with men living with HIV, are at much higher risk, and research into the etiology and pathogenesis of the disease has focused on this group. Other high‐risk groups, such as women with a history of gynecological HPV‐related disease, have been neglected. While single anal cancer lesions typically contain one HPV genotype, multiple genotypes are often detected in anal swabs, complicating attribution. Identifying the causal genotype is essential to assessing the potential impacts of HPV vaccination programs. This study used laser capture microdissection (LCM) to identify the lesion‐specific HPV genotype in anal squamous cell carcinomas from women. Thirty‐five anal cancer samples from women were drawn from the Anal Cancer Outcomes Research Network – Baseline Study (ACORN). Five were excluded, leaving 30 specimens from cisgender women for analysis. p16 immunostaining confirmed high‐risk HPV infection; HPV genotyping using the HPV SPF10‐LiPA25 kit identified the causal genotype for each cancer. The median age at diagnosis was 58.5 (IQR 48–72). All specimens contained a single HPV genotype: 93.3% HPV16, 3.3% HPV18, and 3.3% HPV31. To our knowledge, this is the first study to use LCM on anal cancer biopsies from women to attribute a causal HPV genotype. All specimens tested positive for a single genotype, with HPV16 causing almost all cancers. All cases in this sample could potentially have been prevented by nonavalent prophylactic vaccination, and 97% by the quadrivalent vaccine.

## 1 Introduction

The incidence of anal cancer continues to rise globally [1]. While a rare cancer in the general population, certain groups are at a much higher risk, such as men who have sex with men (MSM), people living with HIV, women with previous human papillomavirus (HPV) related genital disease, such as vulval, vaginal, or cervical cancer or pre‐cancer, and solid organ transplant recipients [2]. MSM living with HIV are at the greatest risk of developing anal cancer [2], and therefore, much of the research into the etiology and pathogenesis of the disease has focused on this group [3]. However, in terms of the overall population, more cases of anal cancer are consistently reported in women compared to men, and are rising [4].

The most common histological type is anal squamous cell carcinoma (ASCC). As with cervical squamous cell cancer, ASCC has been causally linked to persistent oncogenic HPV infection, with ASCCs consistently more likely to be attributed to HPV16. HPV16 and 18 are together responsible for 87% of anal cancer, while the combined contribution of HPV6/11/16/18/31/33/45/52/58 is 96% [5].

Identification of the HPV genotype present within the lesion is critically important to assess potential impacts of prophylactic and/or therapeutic HPV vaccination [6]. Although single anal lesions contain only one HPV genotype [7], multiple genotypes are often detected in anal swabs and from whole tissue sections, making lesion‐level attribution of causality challenging. The presence of HPV in adjacent clinically normal tissue, low‐grade squamous intra‐epithelial lesions and high‐grade squamous lesions (HSIL), may further complicate identification of the genotype present within the cancer itself [8].

Laser capture microdissection (LCM) is a method allowing procurement of a single cell or a group of cells under direct microscope visualization [9]. By utilizing LCM, combined with sensitive genotype testing, the HPV genotype from an individual lesion can be determined [10], and hence causal attribution can be made [11]. Although previous studies have examined the HPV genotypes in cervical and anal high‐grade lesions [8, 12, 13, 14], only one has used LCM on anal cancer specimens, and none have reported lesion‐specific HPV genotypes in women.

We therefore set out to characterize the HPV genotype present within the cancerous lesion of women diagnosed with anal cancer in New South Wales, Australia, using LCM.

## 2 Materials and Methods

### 2.1 Identification of Anal Cancer Cases

This study was conducted at St Vincent's Hospital, Sydney (SVH) (SVH Ethics approval number 07‐058). Samples were drawn from the Anal Cancer Outcomes Research Network – Baseline Study (ACORN study), which collects anal cancer samples from women. The SVH SydPath pathology database was searched for cases of anal cancer in women during the 11‐year study period of August 2009 to August 2018. Medical records were reviewed for each identified case, and data on basic demography, risk factors, medical, anal, and cervical history, anal symptoms, and cancer characteristics were extracted.

### 2.2 Tissue Sectioning and Staining

Archival tissue blocks for each anal cancer case were retrieved from the SVH SydPath archives, and a representative block was chosen in each case. A series of nine sections was cut and processed for histological analysis by using a sandwich sectioning method previously described [10, 15]. Briefly, the two outer sections (sections 1 and 9) were stained with hematoxylin and eosin (H&E) to confirm the initial histological diagnosis of ASCC. The pathologist categorized the SCC as intra‐ or peri‐anal in origin based on the presence of transformation zone tissue (metaplastic squamous epithelial cells or colorectal glands), indicating intra‐anal origin, or keratinization and/or sebaceous glands and/or hair follicles, indicating peri‐anal origin. This classification was recorded for completeness only and was not used to examine differences in HPV genotype between sites. The first inner section (section 2) was stained with CINtec anti‐p16 (Roche, Tucson, Arizona, USA) and counterstained with Gills No. 2 hematoxylin (Australian Biostain, Traralgon, Australia) on the Ventana Benchmark Ultra instrument (Ventana Medical Systems Inc., Oro Valley, Arizona, USA). Sections 1, 2, and 9 were scanned and sent for review by a histopathologist experienced in reading anal biopsy specimens (J. M. R.), to assess p16 positivity and to confirm and annotate the specific lesion for LCM. The second inner section (section 3) was placed onto an ARCTURUS polyethylene naphthalate (PEN) membrane glass slide (Applied Biosystems, Foster City, California, USA) for posterior LCM. Sections 4 and 5 were placed into sterile 1.5 mL microfuge tubes and stored for future DNA, RNA, and protein analysis. Sections 6–8 were mounted on an adhesive glass slide and stored for immunohistochemical staining.

### 2.3 LCM

PEN membrane slides were de‐paraffinized via sequential 5 min incubations in 100% xylene (twice) and 100% ethanol (twice) and then allowed to dry thoroughly prior to LCM. An annotated photograph of the H&E‐stained section was used to guide the selection of abnormal tissue to be cut. Individual cells from a well‐characterized lesion were excised with an ultraviolet cutting laser using the Veritas 704 Laser Capture Microdissection System (Arcturus Bioscience, Mountain View, California, USA). The specific lesion was captured onto an individual CapSure Macro LCM Cap (Applied Biosystems) with an infrared capture laser, labeled, and stored at 4°C until DNA extraction.

### 2.4 DNA Extraction and HPV Detection

DNA was extracted using the PicoPure DNA Extraction Kit (Life Technologies), and 5 µL of the DNA extracted was tested for adequate DNA quantity and quality using a quantitative PCR assay to detect a 110‐bp fragment of the human β‐globin gene as previously reported [16]. Samples were genotyped with the RHA kit HPV SPF10‐LiPA25, version 1 (Labo Bio‐Medical Products BV) to identify HPV6, 11, 16, 18, 31, 33, 34, 35, 39, 40, 42, 43, 44, 45, 51, 52, 53, 54, 56, 58, 59, 66, 68/73, 70, and 74 [17] and also using an RHA kit HPV SPF (Labo Bio‐Medical Products BV) to identify HPV26, 30, 55, 61, 62, c64, 67, 69, 71, 71sub, 82, 83, 84, 85, 87, 89, 90, and 91. HPV 16, 18, 31, 33, 35, 39, 45, 51, 52, 56, 58, 59, 66, and 68 were classified as “high risk”; HPV 26, 34, 53, 67, 69, and 82 were classified as “possible high‐risk.”

## 3 Results

Of the 35 cases initially identified, 3 (9%) were excluded as anal cancer could not be confirmed on histological review. An additional case was excluded as there was inadequate biopsy tissue to analyze. One case was in a transgendered woman, and to avoid reidentification risks, they were omitted from the analysis. Further analysis was therefore conducted on the remaining 30 cases.

### 3.1 Participant Characteristics

Only limited data were available on participants' basic demography, risk factors, medical, anal, and cervical history, anal symptoms, and cancer characteristics. Table 1 summarizes key characteristics of the study population. The median age at diagnosis was 58.5 years (IQR 48–72). Most participants were born in Australia and had an unknown HIV status. Data on smoking status and alcohol consumption were sparse, with most participants having no information on their medical records about these risk factors. Two participants had a previous history of cervical intraepithelial neoplasia, but most had no history recorded. Because these variables were incomplete for most participants, no analysis of associations with HPV genotype was possible.

**Table 1:** Characteristics of cisgender women included in the study.

Characteristic	Total (*N* = 30), *n* (%, 95% CI)
Age at diagnosis of anal cancer (years)	
Median (IQR)	59 (48–72)
Country of birth	
Australia	16 (53.3, 35.1–70.7)
Europe	3 (10.0, 3.1–27.8)
USA	1 (3.3, 0.4–21.6)
Unknown	10 (33.3, 18.5–52.5)
HIV status	
Negative	4 (13.3, 4.9–31.6)
Not recorded/unknown	26 (86.7, 68.4–95.1)
Smoking status	
Non‐smoker	7 (23.3, 11.2–42.4)
Ex‐smoker	4 (13.3, 4.9–31.6)
Smoker	1 (3.3, 0.4–21.6)
Not recorded/unknown	18 (60.0, 41.2–76.3)
Alcohol consumption	
Did not drink alcohol	3 (10.0, 3.1–27.8)
Drank alcohol	8 (26.7, 13.5–45.8)
Not recorded/unknown	19 (63.3, 44.3–78.9)
CIN history	
Previous history	2 (6.5, 1.5–23.5)
Not recorded/unknown	29 (93.5, 76.5–98.5)

### 3.2 Histological Classifications

Twenty‐two (73%) biopsy samples had histological features suggestive of intra‐anal origin, and 8 (27%) of perianal origin.

**Figure 1:**
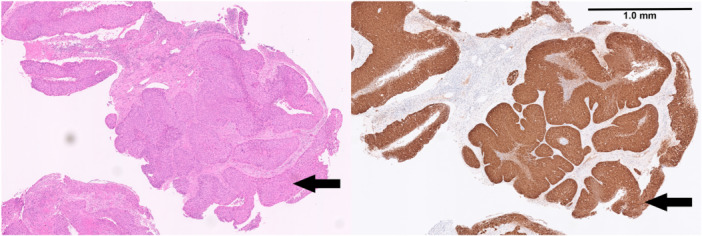
Large nests of intra‐anal squamous cell carcinoma with H&E (left) and p16 (right) immunoperoxidase stains. Overlying the carcinoma, there is a high‐grade squamous intraepithelial lesion (arrowed).

An H&E stain was used to assess cellular morphology. Immunohistochemical staining with p16 was used to identify areas of disrupted cell cycle regulation characteristic of likely HPV‐associated malignancy. All 30 specimens tested positive for p16. Figure 1 shows H&E and p16 staining from the same patient, indicating large nests of intra‐ASCC. An HSIL lesion overlying the carcinoma was also seen (arrows).

### 3.3 LCM and β‐Globin Amplification

**Figure 2:**
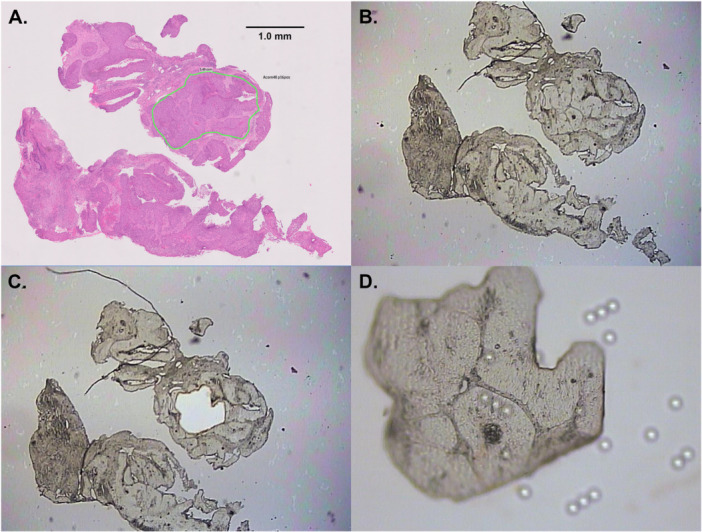
Laser capture microdissection of squamous cell carcinoma of the same patient as Figure 1. (A) Adjacent H&E‐stained biopsy section with SCC annotated by a histopathologist in green; (B) Dewaxed, unstained biopsy section on polyethylene naphthalate membrane slide ready for laser capture microdissection; (C) Small part of the lesion excised with ultraviolet laser, thermoplastic cap heated with infrared laser, and fragments lifted off; (D) Excised fragment ready for DNA extraction and HPV detection.

LCM was performed to isolate cancer cells from the SCC region annotated in green SCC (Figure 2). LCM was applied to the annotated cancerous area in the H&E‐stained section of the same tissue. All samples tested positive for β‐globin amplification, indicating DNA integrity and sample quality.

### 3.4 HPV Genotype Testing

HPV DNA was detected in all specimens (100%), and only one HPV genotype was found in each lesion (Table 2). One (3%) specimen contained HPV31, and one (3%) contained HPV18. HPV16 was detected in the other 28 (93%) cases.

**Table 2:** HPV genotype identified in ASCC lesion.

HPV genotype	Frequency *n* (%, 95% CI)
Any HRHPV	30 (100, –)
HPV16	28 (93, 76–98)
HPV18	1 (3, 0–22)
HPV31	1 (3, 0–22)
Positive p16 dual stain	30 (100%, –)

**Figure 3:**
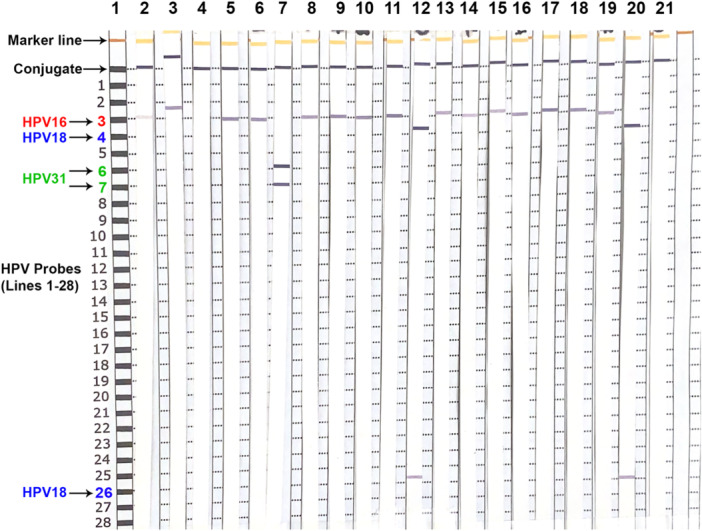
HPV SPF 10‐ LiPA assay. Showing HPV typing of ACORN LCM anal cancer samples and positive and negative controls. Column 1: Guidelines to identify HPV specific probes; Columns 2, 3, 5, 6, 8–11, 13–19: HPV16 positive samples; Column 4: Lung tissue (HPV negative control); Column 7: HPV31 positive sample; Column 12: HPV 18 positive sample; Column 20: HPV18 positive control; Column 21: HPV negative control. HPV31 (probes 6 and 7) and HPV18 (probes 4 and 26) need two probes to be identified.

Figure 3 shows representative results from an HPV SPF10‐LiPA assay. Each run included up to 20 samples (17 clinical specimens, 1 HPV positive, and 2 negative controls), all of which yielded clear HPV‐LiPA signals.

## 4 Discussion

In a sample of 30 cisgender women with ASCC, 100% of biopsies tested positive for HRHPV. Each biopsy contained a single HPV genotype. HPV16 was identified in 93% of cases, while HPV18 and HPV31 were detected in the remaining samples. All anal cancer cases in this study would potentially have been prevented by the nonvalent prophylactic HPV vaccination and 97% by the quadrivalent HPV vaccine.

These results also highlight the importance of working with accurately diagnosed, high‐quality histological material to ensure reliable HPV detection. Although 35 cases were initially identified for inclusion, only 31 met the required diagnostic and quality standards. This aligns with findings from cervical cancer studies, where some HPV‐negative cases were later reclassified following expert pathological review. Similarly, false‐negative HPV results can occur due to poor sample quality or the use of detection methods with suboptimal sensitivity. A recent publication provides guidance on these issues and supports the importance of robust quality assurance for HPV testing [18].

Previous studies aiming to identify causal HPV genotypes in anal cancer specimens have tested small whole‐tissue sections of tumor tissue for HPV DNA and have reported that many tissue samples contain multiple HPV genotypes. The proportion of samples with multiple types detected ranged from 11% to 29%, demonstrating the limitation of using larger tissue samples [7, 19]. Only one other study has used LCM to identify the HPV genotype present in anal cancer specimens, which was performed in men [10]. Four other studies used LCM on anal tissue, but to examine the HPV genotype present in high‐grade lesions rather than cancer, again, all in samples from males. However, all five studies found only one HPV genotype associated with each tissue specimen, consistent with our findings.

LCM allows isolation of a single cancerous lesion and hence eliminates cross‐contamination from HPV types infecting surrounding cells. This was confirmed in our study by the presence of only a single HPV type in all specimens. In our sample group, 100% of samples contained oncogenic HPV types, with almost all (93%) containing HPV16, and the remainder divided between HPV18 and HPV31. This is consistent with HPV16 being the most carcinogenic genotype in the anus [20]. HPV16 prevalence in our study was slightly higher than in the French EDiTH V study, where 86.7% of anal cancer cases in females tested positive for HPV16 [19], and higher than in a global meta‐analysis where 80.7% of anal cancer cases in males and females tested positive for HPV16 [21]. However, direct comparisons with those studies should be made with caution, as they used formalin‐fixed whole‐tissue sections rather than LCM.

The sensitivity of HPV detection in swabs or whole tissue sections, as reported in several anal cancer studies, may be somewhat lower compared to detection using LCM‐isolated tissue. This is likely due to the presence of heterogeneous cell populations in these samples, including cells from nearby non‐cancerous lesions and HPV that may be present without actively replicating. In contrast, LCM‐isolated cancer tissue is composed primarily of tumor cells, allowing for more precise identification of the specific HPV type responsible for the malignancy.

Another important point to consider is that studies analyzing whole tissue sections from anal cancer samples have reported differences in HPV detection according to gender, population characteristics, and age. Some of these studies have shown higher rates of HPV infection in women than in men [21]. In our study, only anal cancer samples from cisgender women were included. Although the number of LCM‐analyzed samples was limited, partly due to the high cost of the LCM technique, we detected single HRHPV infections in 100% of the LCM‐isolated cancer cells. This finding is consistent with LCM facilitating precise molecular characterization, and hence causal attribution, of the HPV genotype within the cancerous lesion [11].

Moreover, we are confident in the reliability of these results, as the risk of cross‐contamination was minimal. Samples were processed on separate days, with only a small number of cases micro‐dissected per day using the LCM instrument. Additionally, all positive and negative controls for DNA extraction, β‐globin analysis, and HPV typing performed as expected.

There were several limitations in the study. There were 2610 females diagnosed with anal cancer in Australia between 2009 and 2018 [22], and we only tested 30 females. Our study, therefore, represents a limited sample in terms of number as well as by location, which meant that statistical analysis was limited. However, the low sample numbers in our study were consistent with all other LCM studies previously published about anal tissue, with sample sizes ranging from 7 to 31 specimens analyzed. Due to the retrospective nature of this study and the archival nature of the specimens, we had only very limited data available for key risk factors for anal cancer, such as history of cervical pre‐cancerous lesions (cervical intraepithelial neoplasia, CIN), HIV status, or pharmacological immunosuppression. This prevented meaningful analysis of risk factors relating to HPV genotype. Cervical and anal HPV infections are highly correlated [23], and women with a history of CIN or living with HIV are known to be at an increased risk of developing anal cancer [2]. Despite this correlation, only a small minority were tested for HIV or had a specifically recorded CIN history.

Prophylactic HPV vaccines could have prevented these anal cancers had these women being vaccinated at a younger age before becoming infected. Given all ACC cases were caused by genotypes targeted by currently licensed vaccines (2v HPV 1618, 4vHPV 611 1618, 9vHPV with additional five oncogenic types of 31, 33, 52 and 58), this strongly supports vaccinating younger populations. In the long term, this could contribute to the elimination of anal cancer.

## Author Contributions

Conceptualization: Richard J. Hillman, Suzanne M. Garland, Monica Molano, and Jennifer M. Roberts. Formal analysis: Clare E. F. Dyer, Suzanne M. Garland, and Monica Molano. Funding acquisition: Alyssa M. Cornall, Suzanne M. Garland, Richard J. Hillman, Jennifer M. Roberts, and Tao Yang. Investigation: Simon Comben, Alyssa M. Cornall, Monica Molano, Gerald L. Murray, Jennifer M. Roberts, and Tao Yang. Methodology: Alyssa M. Cornall, Suzanne M. Garland, Richard J. Hillman, Monica Molano, and Jennifer M. Roberts. Project administration: Simon Comben and Tao Yang. Supervision: Suzanne M. Garland, Richard J. Hillman, and Gerald L. Murray. Validation: Monica Molano and Jennifer M. Roberts. Visualization: Clare E. F. Dyer, Monica Molano, and Jennifer M. Roberts. Writing – original draft: Clare E. F. Dyer and Richard J. Hillman. Writing – review and editing: Simon Comben, Alyssa M. Cornall, Clare E. F. Dyer, Suzanne M. Garland, Richard J. Hillman, Gerald L. Murray, Monica Molano, Jennifer M. Roberts, and Tao Yang.The work reported in the paper has been performed by the authors, unless clearly specified in the text.

## Conflicts of Interest

M. Molano reports a grant from Cancer Council Victoria during the conduct of the study. S. Garland is a member of the Merck Global & Scientific Advisory Boards on HPV vaccines. The other authors declare no conflicts of interest.

## Data Availability

The data that support the findings of this study are available from the corresponding author upon reasonable request.

## References

[1] Y.‐J. Kang , M. Smith , and K. Canfell , “Anal Cancer in High‐Income Countries: Increasing Burden of Disease,” PLoS One 13, no. 10 (2018): e0205105, 10.1371/journal.pone.0205105. 30339668 PMC6195278

[2] G. M. Clifford , D. Georges , M. S. Shiels , et al., “A Meta‐Analysis of Anal Cancer Incidence by Risk Group: Toward a Unified Anal Cancer Risk Scale,” International Journal of Cancer 148, no. 1 (January 2021): 38–47, 10.1002/ijc.33185. 32621759 PMC7689909

[3] M. Lupi , D. Brogden , A. M. Howell , P. Tekkis , S. Mills , and C. Kontovounisios , “Anal Cancer in High‐Risk Women: The Lost Tribe,” Cancers (Basel) 15, no. 1 (December 2022): 60, 10.3390/cancers15010060. 36612055 PMC9817901

[4] A. A. Deshmukh , H. Damgacioglu , D. Georges , et al., “Global Burden of HPV‐Attributable Squamous Cell Carcinoma of the Anus in 2020, According to Sex and HIV Status: A Worldwide Analysis,” International Journal of Cancer 152, no. 3 (February 2023): 417–428, 10.1002/ijc.34269. 36054026 PMC9771908

[5] C. de Martel , M. Plummer , J. Vignat , and S. Franceschi , “Worldwide Burden of Cancer Attributable to HPV by Site, Country and HPV Type,” International Journal of Cancer 141, no. 4 (2017): 664–670. 28369882 10.1002/ijc.30716 PMC5520228

[6] C. L. Trimble , M. P. Morrow , K. A. Kraynyak , et al., “Safety, Efficacy, and Immunogenicity of VGX‐3100, a Therapeutic Synthetic DNA Vaccine Targeting Human Papillomavirus 16 and 18 E6 and E7 Proteins for Cervical Intraepithelial Neoplasia 2/3: A Randomised, Double‐Blind, Placebo‐Controlled Phase 2b Trial,” Lancet 386, no. 10008 (November 2015): 2078–2088, 10.1016/S0140-6736(15)00239-1. 26386540 PMC4888059

[7] R. J. Hillman , S. M. Garland , M. P. W. Gunathilake , et al., “Human Papillomavirus (HPV) Genotypes in an Australian Sample of Anal Cancers,” International Journal of Cancer 135, no. 4 (August 2014): 996–1001, 10.1002/ijc.28730. 24497322

[8] E. T. Callegari , S. N. Tabrizi , J. Pyman , et al., “How Best to Interpret Mixed Human Papillomavirus Genotypes in High‐Grade Cervical Intraepithelial Neoplasia Lesions,” Vaccine 32, no. 32 (July 2014): 4082–4088, 10.1016/j.vaccine.2014.05.041. 24857693

[9] V. Espina , J. D. Wulfkuhle , V. S. Calvert , et al., “Laser‐Capture Microdissection,” Nature Protocols 1, no. 2 (2006): 586–603, 10.1038/nprot.2006.85. 17406286

[10] A. M. Cornall , J. M. Roberts , M. Molano , et al., “Laser Capture Microdissection as a Tool to Evaluate Human Papillomavirus Genotyping and Methylation as Biomarkers of Persistence and Progression of Anal Lesions,” BMJ Open 5, no. 8 (August 2015): e008439, 10.1136/bmjopen-2015-008439. PMC4554896 26310402

[11] M. Molano , S. M. Garland , and A. M. Cornall , “Laser Microdissection for Human Papillomavirus (HPV) Genotyping Attribution and Methylation Pattern Analyses of Squamous Intraepithelial Lesions,” Methods in Molecular Biology 1723 (2018): 167–189, 10.1007/978-1-4939-7558-7_9. 29344860

[12] I. M. Poynten , F. Jin , M. Molano , et al., “Comparison of Four Assays for Human Papillomavirus Detection in the Anal Canal,” Clinical Microbiology and Infection 28, no. 12 (December 2022): 1652.e1–1652.e6, 10.1016/j.cmi.2022.06.027. 35809783

[13] J. M. Roberts , I. M. Poynten , M. Molano , et al., “Human Papillomavirus Genotypes in Anal High‐Grade Squamous Intraepithelial Lesion (HSIL): Anal Intraepithelial Neoplasia Grades 2 (AIN2) and 3 (AIN3) Are Different,” Cancer Epidemiology, Biomarkers & Prevention 29, no. 10 (October 2020): 2078–2083, 10.1158/1055-9965.EPI-20-0664. 32732249

[14] J. van der Marel , J. Berkhof , J. Ordi , et al., “Attributing Oncogenic Human Papillomavirus Genotypes to High‐Grade Cervical Neoplasia: Which Type Causes the Lesion?,” American Journal of Surgical Pathology 39, no. 4 (April 2015): 496–504, 10.1097/PAS.0000000000000342. 25353286

[15] D. A. Machalek , A. E. Grulich , R. J. Hillman , et al., “The Study of the Prevention of Anal Cancer (SPANC): Design and Methods of a Three‐Year Prospective Cohort Study,” BMC Public Health 13 (October 2013): 946, 10.1186/1471-2458-13-946. 24107134 PMC3852594

[16] A. M. Cornall , J. M. Roberts , S. M. Garland , R. J. Hillman , A. E. Grulich , and S. N. Tabrizi , “Anal and Perianal Squamous Carcinomas and High‐Grade Intraepithelial Lesions Exclusively Associated With “Low‐Risk” HPV Genotypes 6 and 11,” International Journal of Cancer 133, no. 9 (November 2013): 2253–2258, 10.1002/ijc.28228. 23616200

[17] A. M. Cornall , W. H. Quint , S. M. Garland , and S. N. Tabrizi , “Evaluation of an Automated SPF10‐LiPA25 Assay for Detection and Typing of Human Papillomavirus in Archival Samples,” Journal of Virological Methods 199 (April 2014): 116–118, 10.1016/j.jviromet.2014.01.012. 24487100

[18] J. L. Pretet , L. S. Arroyo Muhr , K. Cuschieri , et al., “Human Papillomavirus Negative High Grade Cervical Lesions and Cancers: Suggested Guidance for HPV Testing Quality Assurance,” Journal of Clinical Virology 171 (April 2024): 105657, 10.1016/j.jcv.2024.105657. 38401369 PMC11863830

[19] L. Abramowitz , A. C. Jacquard , F. Jaroud , et al., “Human Papillomavirus Genotype Distribution in Anal Cancer in France: The EDiTH V Study,” International Journal of Cancer 129, no. 2 (July 2011): 433–439, 10.1002/ijc.25671. 20839262

[20] C. Lin , S. Franceschi , and G. M. Clifford , “Human Papillomavirus Types From Infection to Cancer in the Anus, According to Sex and HIV Status: A Systematic Review and Meta‐Analysis,” Lancet Infectious Diseases 18, no. 2 (February 2018): 198–206, 10.1016/S1473-3099(17)30653-9. 29158102 PMC5805865

[21] L. Alemany , M. Saunier , I. Alvarado‐Cabrero , et al., “Human Papillomavirus DNA Prevalence and Type Distribution in Anal Carcinomas Worldwide: HPV in Anal Cancers,” International Journal of Cancer 136, no. 1 (January 2015): 98–107, 10.1002/ijc.28963. 24817381 PMC4270372

[22] Australian Institute of Health and Welfare (AIHW) , Cancer Data in Australia (2024).

[23] C. Lin , J. Slama , P. Gonzalez , et al., “Cervical Determinants of Anal HPV Infection and High‐Grade Anal Lesions in Women: A Collaborative Pooled Analysis,” Lancet Infectious Diseases 19, no. 8 (2019): 880–891, 10.1016/S1473-3099(19)30164-1. 31204304 PMC6656696

